# Associations Between Physical Activity Intensity, Resilience, Self-Esteem and Health-Related Quality of Life in University Students: A Structural Equation Modeling Approach

**DOI:** 10.3390/healthcare14111438

**Published:** 2026-05-22

**Authors:** Zhangyu Yang, Gracia Cristina Villodres, Jianfei Ye, Xing Zhang, Li Huang, José Joaquín Muros

**Affiliations:** 1 Department of Didactics of Body Expression, Faculty of Educational Sciences, University of Granada, 18071 Granada, Spain; yangzhangyu@correo.ugr.es (Z.Y.); jjmuros@ugr.es (J.J.M.); 2 College of Physical Education, Fuyang Normal University, Fuyang 236037, China; 3 Institute of Physical Education, Huangshan University, Huangshan 245041, China; 600077@hsu.edu.cn; 4 Department of Physical Education and Sport, Faculty of Sport Sciences, University of Granada, 18071 Granada, Spain; starz@correo.ugr.es; 5 Institute of Sports Science, College of Physical Education, Southwest University, Chongqing 400715, China; hl2021@email.swu.edu.cn

**Keywords:** physical activity, exercise intensity, resilience, self-esteem, health-related quality of life, university students

## Abstract

**Background**: University students often face significant psychological challenges and lifestyle disruptions that may compromise their mental resources and health-related quality of life (HRQoL). Although associations between physical activity (PA) and mental health have been widely reported, few studies have integrated different PA intensities, sedentary behavior, and psychological resources jointly related in one analytical model. **Objective**: This study investigated the relationships among vigorous (VPA), moderate (MPA), and light (LPA) physical activity, sedentary behavior (SB), resilience (RES), self-esteem (SE), and HRQoL in a sample of Chinese university students. **Methods**: A cross-sectional survey included 1560 university students from six universities in China, with a mean age of 19.43 ± 1.15 years; the sample comprised 434 males (27.8%) and 1126 females (72.2%). Relationships among the variables were tested using path analysis within a structural equation modeling framework. **Results**: Greater PA engagement was related to higher RES, SE, and HRQoL, whereas SB was not significantly associated with RES. All three PA intensities were positively associated with RES, although the magnitude of these associations varied. In addition, RES was also related to higher SE and HRQoL, and SE was related to higher HRQoL. **Conclusions**: These findings suggest that PA is associated with psychological resources and HRQoL among university students. Longitudinal and intervention studies are needed to determine the directionality and mechanisms underlying these relationships.

## 1. Introduction

Psychological distress among university students has become an increasingly important public health concern, particularly due to the rising prevalence of anxiety and depressive symptoms reported in this population [1]. The university stage is often characterized by substantial emotional, behavioral, and social changes, which may increase students’ vulnerability to psychological difficulties [2]. Such challenges are particularly pronounced when adjusting to life away from home, managing academic tasks, navigating interpersonal relationships and establishing healthy lifestyle routines, with all of this coinciding with the ongoing development of self-regulation skills. The accumulation of these stressors may reduce participation in healthy behaviors and weaken psychological resources, which may compromise students’ health-related quality of life (HRQoL) [3,4].

Concerns regarding university students’ mental health and quality of life encourage greater attention to the psychological resources that may support adjustment during this developmental period. Resilience and self-esteem are particularly relevant in this context because they are closely related to the way in which students interpret stress and manage adversity, which, subsequently, contributes to improved HRQoL [5,6]. For example, students with stronger self-esteem tend to report more adaptive responses to academic pressure, greater feelings of self-worth and belonging, and lower loneliness, all of which are associated with better mental health and HRQoL [7]. Resilience is also important for students’ adaptation to university life. Emotional regulation, perceived social support, and a sense of fit with the university environment may help students manage academic stress and maintain subjective well-being [8,9]. In addition, resilience and self-esteem appear to be closely connected, suggesting that students with stronger resilience may also exhibit more stable self-appraisals and emotional functioning [10,11]. Together, these psychological resources may provide a useful foundation for understanding student well-being in higher education settings.

Beyond individual psychological traits, physical activity (PA) is increasingly recognized as a safe and effective non-pharmacological strategy for promoting the development and enhancement of psychological resources [12]. Previous research has indicated that regular PA is associated with lower stress reactivity, better physical and mental well-being, and more adaptive responses to stress [13,14]. Engagement in PA may additionally expose individuals to socially interactive and challenging environments that support the development of resilience. Sustained participation in demanding physical activities has been linked to attentional control, cognitive flexibility, and social skills, which are important capacities for adapting to changing environments and coping with psychological challenges [15].

Recent studies further indicate that the psychological benefits of PA may vary according to intensity. Some intervention studies have reported improvements in resilience following moderate-intensity aerobic exercise, high-intensity interval training, or moderate-intensity training [16,17]. Other cross-sectional evidence has suggested that vigorous PA may be more closely related to resilience than moderate-intensity activity in certain student samples [18]. Nevertheless, the psychological impact of the intensity of PA engagement remains inconclusive, highlighting the need for further comparative and integrative investigation.

Although PA, sedentary behavior, resilience, self-esteem, and HRQoL have been examined in previous studies, these variables have often been considered separately. Limited research has integrated multiple PA intensities, sedentary behavior, and psychological resources within a single analytical framework among university students. To address this gap, the present study investigated the relationships among vigorous PA (VPA), moderate PA (MPA), light PA (LPA), sedentary behavior (SB), resilience (RES), self-esteem (SE), and HRQoL in Chinese university students using path analysis within an SEM framework. The following hypotheses were developed from prior evidence:

(1) PA will be positively associated with RES;

(2) RES will be positively associated with SE and HRQoL;

(3) SB will show weaker or non-significant associations with RES.

## 2. Materials and Methods

### 2.1. Participants and Procedures

Data were collected in December 2024 through a cross-sectional online survey of undergraduate students enrolled at six comprehensive universities in Anhui Province, China. Before data collection, the research team reviewed the relevant literature to select appropriate measurement instruments. The questionnaire was then prepared using the Sojump online survey platform (Changsha Ranxing Information Technology Co., Ltd., Changsha, China; www.sojump.com, accessed on 1 December 2024). A digital recruitment poster containing brief study information and a QR code linking to the questionnaire was then prepared for online distribution.

During the formal data collection phase, 21 university instructors were invited to assist with survey dissemination. These instructors shared the digital poster with their students via group chats on QQ and WeChat (Tencent Holdings Ltd., Shenzhen, China), as well as through in-person communication channels. In order to minimize response bias, participants were not informed of the study’s specific hypotheses or expected relationships between variables. Prior to completing the survey, all participants provided informed consent, which emphasized the voluntary nature of participation and explained that all gathered data would be kept confidential and anonymous. Participants were also informed that they could withdraw at any time throughout the study process. No monetary or material incentives were offered for participation. The present study was conducted in accordance with the Declaration of Helsinki and was approved by the Ethics Review Committee of Southwest University (SWUETH20230912003).

In order to ensure data quality, four attention-check items were included in the questionnaire to detect inattentive or careless responding. During data cleaning, questionnaires were excluded if they contained logical inconsistencies, incomplete responses, or failed any of the verification items. After screening, 1560 valid responses were included in the final analysis, comprising 434 males and 1126 females. Detailed participant characteristics are presented in Table 1.

### 2.2. Instruments and Measurements

#### 2.2.1. IPAQ-SF

PA was assessed using the International Physical Activity Questionnaire–Short Form (IPAQ-SF), a seven-item self-report tool for individuals aged 15–69 years. The questionnaire records the number of days and minutes spent walking and engaging in moderate- and vigorous-intensity activities during the previous seven days [19]. Standard MET coefficients were applied to each activity category: 3.3 for walking, 4.0 for moderate activity, and 8.0 for vigorous activity. Weekly PA was then calculated by multiplying MET value, frequency, and duration, and expressed as MET-minutes/week. Based on these values, participants are categorized as engaging in low, moderate, or high levels of PA [20]. The IPAQ-SF also captures average daily sitting time and has been validated for use with Chinese university students [21]. MET-min/week was used for group comparisons, whereas minutes of each PA intensity were entered into the path model.

#### 2.2.2. The Connor-Davidson Resilience Scale

The Connor-Davidson Resilience Scale (CD-RISC) is a 25-item self-report instrument that assesses psychological resilience using a five-point Likert scale (0 = “Not at all” to 4 = “Most of the time”). The scale comprises five dimensions: (1) Persistence–Tenacity–Self-efficacy; (2) Control under pressure; (3) Adaptability and support networks; (4) Control and purpose; and (5) Spirituality [22]. Overall resilience scores are obtained by summing all individual subscale scores, with higher total scores indicating greater resilience. The scale has been validated for use with Chinese university students [23]. In this study, internal consistency was high (Cronbach’s α = 0.957).

#### 2.2.3. The Rosenberg Self-Esteem Scale

Self-esteem was assessed using the 10-item Rosenberg Self-Esteem Scale (RSES), which includes both positively and negatively worded items [24]. Responses were scored on a four-point Likert scale. Positive items were scored from 4 (“Strongly agree”) to 1 (“Strongly disagree”), whereas negative items were reverse-coded. The RSES has been validated in Chinese university student samples [25]. In the present sample, the RSES demonstrated good internal consistency (Cronbach’s α = 0.896).

#### 2.2.4. SF-36

The 36-Item Short Form Health Survey (SF-36) is a widely used instrument for assessing HRQoL. It comprises 36 items with scores ranging from 0 to 100. Overall HRQoL scores are calculated by summing individual item scores (taking into account previous reverse item conversion), with higher scores indicating better self-reported health. The SF-36 has been validated among Chinese university students [26]. In the present sample, the SF-36 demonstrated excellent internal consistency (Cronbach’s α = 0.903).

### 2.3. Data Analysis

Data were analyzed using IBM SPSS 26.0. Descriptive statistics are presented as means and standard deviations. Before conducting group comparisons, the distributions of the main variables were inspected using skewness and kurtosis. The values fell within commonly accepted ranges for the main variables, supporting the use of parametric analyses. Given the large sample size, these tests were considered appropriate for the present analyses [27]. Independent-sample *t*-tests were used for two-group comparisons, while one-way ANOVA followed by Tukey post hoc tests was applied for comparisons across three PA groups. Effect size was reported in line with Cohen’s d and interpreted as small (d = 0.2), moderate (d = 0.5) or large (d = 0.8) [28].

**Figure 1:**
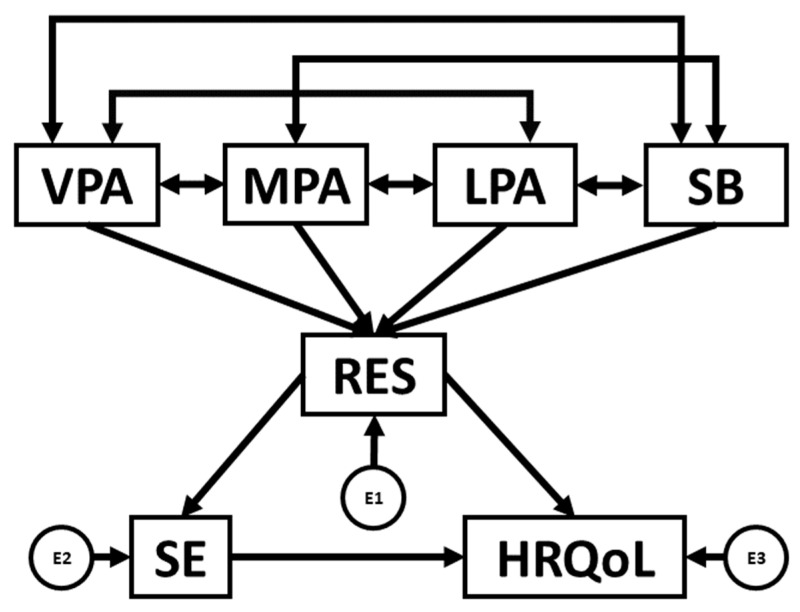
Structural equation model. VPA: vigorous physical activity; MPA: moderate physical activity; LPA: light physical activity; SB: sedentary behavior; RES: resilience; SE: self-esteem; HRQoL: health-related quality of life. E1–E3: error terms. ←: directional relationships between variables; ↔: correlation between two variables.

Path analysis within a structural equation modeling framework was performed in AMOS using seven observed variables: VPA, MPA, LPA, SB, RES, SE, and HRQoL. In the model, VPA, MPA, LPA, and SB were specified as exogenous variables, while RES, SE, and HRQoL were treated as endogenous variables (Figure 1). Given that the primary aim of this study was to examine structural relationships among variables rather than to test measurement models, composite scores were used instead of latent constructs.

Model adequacy was assessed using several fit statistics, including χ^2^, CFI, IFI, NFI, TLI, and RMSEA. Values greater than 0.90 for CFI, IFI, NFI, and TLI were considered acceptable, and values above 0.95 indicated excellent fit. For RMSEA, values below 0.08 were interpreted as acceptable and values below 0.05 as a good fit [29].

## 3. Results

Descriptive characteristics of the study sample are presented in Table 2. Compared with women, men showed higher scores for PA, RES, SE, and HRQoL (all *p* < 0.001), with small-to-moderate effect sizes. Regarding sibling status, students with one or more siblings demonstrated significantly higher RES than those without siblings (*p* = 0.006; small effect size).

As shown in Table 3, significant differences were observed across PA groups for RES, SE, and HRQoL. Students in the high PA group reported significantly higher RES compared to both moderate and low PA groups (all *p* < 0.001), while the moderate group also showed higher RES than the low PA group (*p* = 0.002). A similar pattern was observed for SE, with both high and moderate PA groups reporting significantly higher scores than the low PA group (all *p* < 0.001). However, no significant difference was found between the moderate and high PA groups. For HRQoL, students in the high PA group reported significantly higher scores than those in the low and moderate PA groups (*p* < 0.001 and *p* = 0.006, respectively), whereas no significant difference was observed between the moderate and low PA groups.

**Figure 2:**
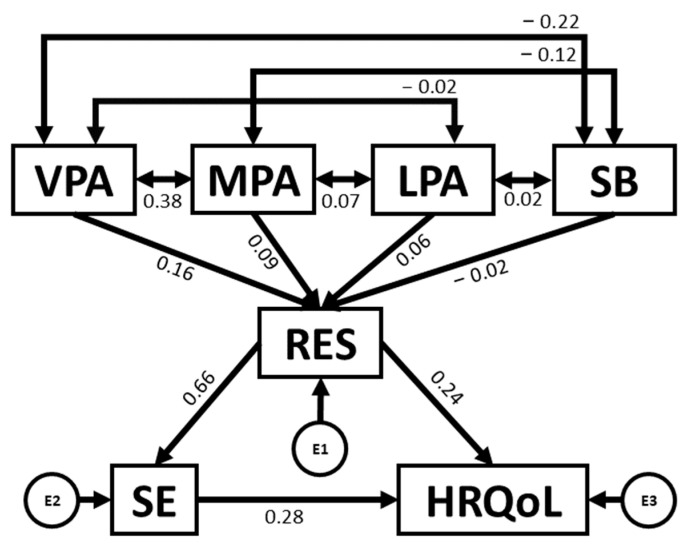
Structural equation model for the overall sample. Note. VPA: vigorous physical activity; MPA: moderate physical activity; LPA: light physical activity; SB: sedentary behavior; RES: resilience; SE: self-esteem; HRQoL: health-related quality of life; E1–E3: error terms. ←: directional relationships between variables; ↔: correlation between two variables.

Path analysis results are shown in Table 4 and Figure 2. Overall model fit was good (χ^2^ = 21.4, df = 8, *p* = 0.006; CFI = 0.992; IFI = 0.992; TLI = 0.980; NFI = 0.988; RMSEA = 0.033). In terms of the relationships between PA variables, VPA was positively associated with MPA and negatively associated with SB (both *p* < 0.001), but was not significantly related to LPA. MPA showed a positive relationship with LPA and a negative relationship with SB (*p* < 0.01), while LPA was not significantly related to SB. With respect to the main pathways, VPA, MPA, and LPA were all positively associated with RES (all *p* < 0.05), whereas SB showed no significant relationship with RES. RES was related to higher SE and HRQoL (both *p* < 0.001), and SE was related to higher HRQoL (*p* < 0.001).

## 4. Discussion

The present study examined the associations among PA, psychological resources, and health-related quality of life (HRQoL) in a sample of university students. Male students scored higher than female students on PA, RES, SE, and HRQoL. In addition, higher levels of PA engagement were associated with RES, SE, and HRQoL. With respect to the structural relationships, all three intensities of PA were positively associated with RES, although the magnitude of these associations varied across intensity levels. In contrast, sedentary behavior (SB) was not significantly related to resilience. Furthermore, RES was positively associated with both SE and HRQoL, while SE was also positively associated with HRQoL.

### 4.1. Sociodemographic Variations in PA, RES, SE and HRQoL

Sex-based differences were observed across several variables, with male students showing higher PA, RES, SE, and HRQoL scores. This result aligns with earlier evidence that male students generally report greater participation in moderate-to-vigorous PA than female students [30]. From the perspective of social role theory, men are often encouraged to display energy, competitiveness and adventurousness, whilst women are more frequently expected to be gentle and reserved [31]. Such gendered expectations may contribute to differences in exercise motivation and self-efficacy, which in turn may be associated with higher levels of PA participation among males [32].

Previous meta-analytical findings have similarly suggested gender-related differences in resilience, suggesting that men tend to score higher on measures related to stress management and emotional regulation. Greater PA engagement, particularly at higher intensities, may be linked to adaptive psychological responses [33,34]; however, this interpretation should be considered cautiously given the cross-sectional design.

Regarding SE and HRQoL, the present findings indicate higher scores among male students, although existing literature reports mixed evidence on gender differences in these variables [35,36]. These inconsistencies may reflect the combined influence of behavioral, psychological, and sociocultural factors. In contexts where males participate more actively in sports, they may receive more positive psychological feedback in terms of coping with academic stress, emotional regulation and life satisfaction, thereby contributing to elevated self-evaluations and perceived quality of life [7].

In addition to gender differences, students with siblings reported significantly higher RES than those without siblings. Earlier research has similarly indicated that sibling interactions during early development may be associated with the development of interpersonal skills, emotional regulation, and coping abilities, which are closely linked to resilience [37,38].

### 4.2. Associations Between PA Level, RES, SE and HRQoL

The present study also examined differences in RES, SE, and HRQoL across varying levels of PA. Overall, students with higher levels of PA tended to report more favorable outcomes across all three variables. Specifically, students in the high PA group reported higher levels of RES compared to those in the moderate and low PA groups. Comparable findings have been reported in previous research examining that engagement in PA, particularly at moderate-to-higher intensities, may be associated with improved emotional regulation and adaptive coping capacities [39]. However, given the cross-sectional design and the use of self-reported PA measures, these findings should be interpreted with caution.

A similar pattern was observed for SE, with both moderate and high PA groups showing higher levels than the low PA group. These findings may indicate that participation in PA is associated with more positive self-evaluations [40,41]. Notably, the moderate and high PA groups did not differ significantly, which may indicate that the association between PA and SE does not increase linearly across intensity levels.

With regard to HRQoL, students who engage in high amounts of PA reported significantly higher scores than those who engage in only moderate and low amounts. Greater PA engagement also corresponded to more favorable perceptions of physical functioning and overall well-being, possibly through links with emotional experience and psychological resources [40].

Overall, the present findings indicate that PA engagement is positively associated with psychological resources and HRQoL, although the magnitude of these associations may vary across different levels of activity.

### 4.3. Pathways Linking PA, RES, SE and HRQoL

In the structural path model, VPA, MPA and LPA were each positively associated with RES, whereas SB was not significantly related to RES. The absence of a significant SB–RES association is consistent with earlier findings [34] and may partly reflect the use of sitting time as a single indicator of SB, which may not adequately represent the different forms, domains and contexts of this behavior. From the perspective of the cross-stressor adaptation hypothesis, regular PA may be linked to physiological adaptation and lower reactivity to everyday stressors [42], providing a possible explanation for the association between PA and RES [43]. However, the observed differences across PA intensities should be interpreted cautiously, particularly because PA was assessed using self-report measures. Although previous studies have suggested that higher-intensity activity may be related to emotional regulation and coping capacity in stressful contexts [16,18], longitudinal and experimental studies are needed to determine whether such intensity-related patterns are stable and clinically meaningful.

The associations between RES, SE and HRQoL could also be interpreted within broader resilience and self-regulation frameworks. Resilience may support psychological stability by helping individuals mobilize cognitive, emotional and behavioral resources when facing stress or adversity [44]. This interpretation is compatible with the challenge model, which suggests that adaptive responses to adversity may develop through effective self-regulation processes [45]. Previous research has also indicated that resilience may be linked to lower stress vulnerability and better psychological functioning through several pathways, including buffering risk exposure, interrupting negative response patterns, strengthening psychological resources, and supporting adaptive coping [46,47]. In the present study, SE was also positively associated with HRQoL, suggesting that students with more favorable self-appraisals may perceive themselves to have better well-being. This finding is consistent with the risk-buffering model, which proposes that self-esteem may help reduce the psychological impact of negative experiences [48].

Finally, significant associations were observed among different PA intensities, particularly between VPA and MPA. This may indicate that students often engage in multiple forms of activity rather than participating in a single intensity category. Previous studies have similarly reported that combinations of moderate- and vigorous-intensity activity are commonly used to meet PA recommendations [34]. Thus, future research and university health promotion efforts may benefit from considering PA as a multidimensional behavior rather than focusing exclusively on a single intensity level.

### 4.4. Limitations and Future Directions

Although the present study contributes to understanding the associations among PA intensity, SB, RES, SE and HRQoL in university students, several limitations need to be considered. First, the cross-sectional design prevents conclusions about causality or temporal ordering. Longitudinal and experimental studies are needed to test the stability of these findings and to clarify the mechanisms underlying the observed associations. Second, all variables were collected through self-report questionnaires at a single time point. Thus, findings may be affected by recall, social desirability and common-method biases, which may have inflated the observed associations between variables. In addition, SB was assessed using only one indicator (i.e., sitting time), which may not fully capture the different domains, patterns and contexts that characterize sedentary behavior. Third, the path model was based on composite scores rather than latent constructs. As a result, measurement error could not be explicitly modeled, which may have reduced the precision of the estimated structural relationships. Future studies using latent-variable SEM could provide a more refined evaluation of both measurement and structural components. Fourth, participants were recruited from universities in central China, and the sample included a higher proportion of female students. These features may restrict the generalizability of the findings and should be considered when interpreting sex-related differences. Future studies should include samples from more diverse regions, cultural contexts and gender distributions. Fifth, several potentially important confounding variables, including body mass index (BMI), socioeconomic status, health status, field of study, sleep patterns, and academic stress, were not controlled for in the present study, which may have influenced the observed relationships. Future research should incorporate these factors and further explore the complexity of associations between PA and psychological outcomes, including potential non-linear patterns. In addition, the present study did not assess whether the proposed structural relationships are consistent across different subgroups (e.g., sex or academic year). Future studies employing multi-group structural equation modeling are warranted to evaluate the stability and generalizability of the model.

## 5. Conclusions

The present exploratory path analysis suggests that different intensities of PA are associated with psychological resources and HRQoL among university students. All three levels of PA engagement were positively associated with RES, which was, in turn, positively associated with both SE and HRQoL. These findings may provide preliminary support for considering PA within university-based health promotion and student well-being initiatives. However, further longitudinal and experimental studies are needed before practical recommendations regarding PA intensity can be established.

## Figures and Tables

**Table 1:** Demographic characteristics of the sample (*n* = 1560).

Characteristics	*n*	Percents (%)
*Sex*		
Male	434	27.8%
Female	1126	72.2%
*Siblings*		
No Siblings	1220	78.2%
One or more siblings	340	21.8%
*Academic area*		
Physical Education	248	15.9%
Education Sciences	556	35.6%
Health Sciences	143	9.2%
Other Non-health Sciences	613	39.3%
*Grade*		
Freshman	869	55.7%
Sophomore	620	39.7%
Other grades	71	4.6%

**Table 2:** Group differences in PA, RES, SE, and HRQoL by sex and sibling status.

Grouping Variable	Group	PA	RES	SE	HRQoL
		(MET-min/Week)			
Sex	Men	4240.25 ± 2871.77	66.21 ± 16.53	31.05 ± 4.71	624.48 ± 106.79
	(*n* = 434)				
	Women	2592.92 ± 1977.93	61.22 ± 14.48	30.18 ± 4.79	586.84 ± 110.41
	(*n* = 1126)				
	*p* value	<0.001	<0.001	<0.001	<0.001
	Cohen’s d	0.668	0.321	0.183	0.344
Siblings	No	3016.05 ± 2292.10	62.05 ± 14.93	30.31 ± 4.71	597.57 ± 109.97
	Siblings				
	(*n* = 1220)				
	One or more	3117.37 ± 2666.90	64.60 ± 16.17	30.84 ± 5.02	596.41 ± 113.33
	sibling				
	(*n* = 340)				
	*p* value	0.068	0.006	0.865	0.310
	Cohen’s d	0.041	0.164	0.109	0.010

Note. RES: resilience; SE: self-esteem; HRQoL: health-related quality of life. Effect sizes are interpreted as small (d = 0.2), medium (d = 0.5), and large (d = 0.8).

**Table 3:** Differences in RES, SE, and HRQoL according to PA levels.

	PA Levels	M	SD	Comparison	*p* Value	95% CI
	(MET-min/Week)					
RES	Low (*n* = 518)	59.00	14.30	Low vs. Moderate	0.002	[−5.40, −1.03]
				Low vs. High	<0.001	[−9.69, −5.36]
	Moderate (*n* = 512)	62.21	14.57	Moderate vs. High	<0.001	[−6.48, −2.14]
	High (*n* = 530)	66.52	15.86			
SE	Low (*n* = 518)	29.44	4.76	Low vs. Moderate	<0.001	[−1.83, −0.45]
				Low vs. High	<0.001	[−2.47, −1.11]
	Moderate (*n* = 512)	30.58	4.73	Moderate vs. High	0.067	[−1.34, 0.03]
	High (*n* = 530)	31.23	4.68			
HRQoL	Low (*n* = 518)	518.88	113.49	Low vs. Moderate	0.161	[−28.58, 3.54]
				Low vs. High	<0.001	[−49.25, −17.40]
	Moderate (*n* = 512)	594.40	108.95	Moderate vs. High	0.006	[−36.78, −4.83]
	High (*n* = 530)	615.21	107.16			

Note. RES: resilience; SE: self-esteem; HRQoL: health-related quality of life.

**Table 4:** Regression weights for the overall sample.

Association Between			RW				SRW
Variables			Estimation	SE^2^	CR	*p*	Estimation
RES	←	VPA	0.013	0.002	5.952	<0.001	0.162
RES	←	MPA	0.006	0.002	3.525	<0.001	0.094
RES	←	LPA	0.003	0.001	2.425	0.015	0.060
RES	←	SB	0.000	0.000	−0.747	0.455	−0.019
SE^1^	←	RES	0.207	0.006	34.689	<0.001	0.660
HRQoL	←	RES	1.761	0.215	8.179	<0.001	0.242
HRQoL	←	SE	6.477	0.687	9.433	<0.001	0.280
MPA	↔	VPA	16,859.534	1197.421	14.080	<0.001	0.382
MPA	↔	LPA	5054.311	1910.342	2.646	0.008	0.067
VPA	↔	LPA	−1263.659	1671.250	−0.756	0.450	−0.019
SB	↔	LPA	1038.462	1432.686	0.725	0.469	0.018
SB	↔	VPA	−7436.072	861.584	−8.631	<0.001	−0.224
SB	↔	MPA	−4490.352	965.772	−4.649	<0.001	−0.119

Note. VPA: vigorous physical activity; MPA: moderate physical activity; LPA: light physical activity; SB: sedentary behavior; RES: resilience; SE^1^: self-esteem; HRQoL: health-related quality of life; RW: regression weight; SRW: standardized regression weight; SE^2^: standard error; CR: critical ratio; ←: directional relationships between variables; ↔: correlation between two variables.

## Data Availability

The data that support the findings of this study are available from the corresponding author upon reasonable request.

## Abbreviations

The following abbreviations are used in this manuscript:

HRQoL	Health-related quality of life
PA	Physical activity
VPA	Vigorous physical activity
MPA	Moderate physical activity
LPA	Light physical activity
SB	Sedentary behavior
RES	Resilience
SE	Self-esteem
SEM	Structural equation modeling
